# Sexual health and wellbeing among female pelvic cancer survivors following individualized interventions in a nurse-led clinic

**DOI:** 10.1007/s00520-022-07294-x

**Published:** 2022-08-05

**Authors:** Linda Åkeflo, Gail Dunberger, Eva Elmerstig, Viktor Skokic, Gunnar Steineck, Karin Bergmark

**Affiliations:** 1 Division of Clinical Cancer Epidemiology, Department of Oncology, Institute of Clinical Science, The Sahlgrenska Academy, University of Gothenburg, 413 45, Gothenburg, Sweden; 2 Ersta Bräcke Sköndal, University College, Stockholm, Sweden; 3 Centre for Sexology and Sexuality Studies, Department of Social Work, Malmö University, Malmö, Sweden; 4 Department of Molecular Medicine and Surgery, Karolinska Institutet, Stockholm, Sweden; 5 Department of Pelvic Cancer, Karolinska University Hospital, Stockholm, Sweden

**Keywords:** Nurse, Cancer survivors, Physical side effects, Rehabilitation, Sexual dysfunction, Radiotherapy

## Abstract

**Purpose:**

Treatment-induced sexual and intestinal dysfunctions coexist among women after pelvic radiotherapy. We aimed to explore if sexual health and wellbeing may be improved after radiotherapy following nurse-led interventions and if an association exists between improved intestinal health and sexual health.

**Methods:**

A population-based cohort of women treated with pelvic radiotherapy underwent interventions at a nurse-led clinic at Sahlgrenska University Hospital, Sweden, from 2011 to 2017. Self-reported questionnaires were used, pre- and post-intervention, to compare self-reported changes in sexual health and wellbeing. A regression model was performed to explore the association between intestinal and wellbeing variables.

**Results:**

Among the 260 female pelvic cancer survivors included in the study, more women reported increased than decreased satisfaction with overall sexual health post-intervention (26.0% vs. 15.3%, *p* = 0.035). They also reported significantly reduced superficial genital pain (25.8% vs. 13.1%, *p* ≤ 0.025), reduced deep genital pain (23.1% vs. 8.0%, *p* ≤ 0.001), increased QoL (42.7% vs. 22.4%, *p* < 0.001), and reduced levels of depression (43.1% vs. 28.0%, *p* = 0.003) or anxiety (45.9% vs. 24.4%, *p* < 0.001) post-intervention. We found a significant association between reduced urgency to defecate and improved satisfaction with overall sexual health (RR 3.12, CI 1.27–7.68, *p* = 0.004) and between reduced urgency to defecate with fecal leakage and reduced anxious mode (RR 1.56, CI 1.04–2.33, *p* = 0.021).

**Conclusion:**

Sexual health and wellbeing can be improved by interventions provided in a nurse-led clinic focusing on physical treatment-induced late effects. Further research to optimize treatment strategies in female pelvic cancer survivors is needed.

## Introduction

Sexual dysfunction is highly prevalent among female cancer survivors treated with pelvic radiotherapy [1–3] which, not infrequently, leads to chronic problems affecting their quality of life [4, 5]. With an estimated 4700 new pelvic cancer diagnoses every year in Sweden, pelvic cancer survivorship care needs to be strengthened [6]. For women treated with pelvic radiotherapy, long-term sexual dysfunction is a common and distressing problem regardless of specific pelvic cancer diagnosis [7–9]. In recent decades, efforts have been made when planning radiotherapy to avoid and treat radiotherapy-induced late effects, including sexual dysfunction, intestinal- and urinary tract dysfunctions, and lymphedema [3, 10–12]. Commonly, cancer and cancer treatment negatively affect sexual function and satisfaction due to direct and indirect physiological, psychological, and interpersonal factors [4, 5, 13]. Previous studies have suggested a broad scope of interventions and simple strategies addressing sexual health concerns to enable sexual rehabilitation after cancer treatment [14–17].

The importance of sexual health may vary with age and sexual activity, and relates to general health. For many women with chronic illnesses, sexual health remains important despite other symptoms [13]. Since radiotherapy comprises a wide range of physical problems, multidimensional interventions directed at female pelvic cancer survivors are considered to offer a solution to the widespread issue of decreased health after completion of pelvic radiotherapy [18]. However, it is not known whether sexual health problems could improve in female pelvic cancer survivors following a biopsychosocial-approached nurse-led intervention.

Previous research in women with non-malignant diseases reports associations between impaired intestinal function and sexual health [19, 20]. In women with a history of gynecological cancer, the presence of intestinal late effects, such as fecal leakage and loose stools, is reported to severely impair quality of life [21, 22]. Living with fecal incontinence may lead to the avoidance of being in or embarking on relationships that might lead to sexual intimacy [23]. Studies focusing on interventions aimed at improving female pelvic cancer survivors’ sexual health have been mainly limited to small prospective studies and pilot randomized controlled trials [24, 25]. There are therefore calls for studies with an intervention focus to fill the gaps [26–28].

In this paper, we use data from a population-based cohort of female cancer survivors treated with pelvic radiotherapy who had completed interventions in a nurse-led clinic, as previously described [29]. We aim to study whether sexual health and wellbeing improves among women treated with radiotherapy following a nurse-led intervention and to explore associations between improved sexual and intestinal health.

## Methods

The cohort profile, the data collection, the questionnaire, and interventions for the current study can be found outlined elsewhere [29]. This section provides a brief summary.

### Setting and study participants

All women receiving radiotherapy to the pelvic region with curative intent during 2007–2016 at Sahlgrenska University Hospital in Sweden, and pelvic cancer survivors referred to the nurse-led clinic, were invited for inclusion in the study. The women included in the analysis for this study had participated in a nurse-led clinic intervention and completed baseline and follow-up questionnaires.

### Data collection

**Fig. 1:**
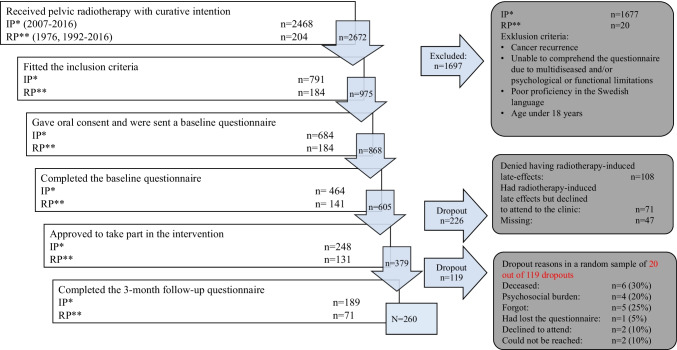
Flow-chart of recruitment and participation in the baseline- and 3-month follow-up questionnaires, numbers (*n*) and reasons for dropouts among 20 out of 119 randomized drop-outs. The total response rate among the female pelvic cancer survivors who agreed to visit the clinic was 260 of 379 (68.6%). *Inventoried patients, **Referred patients

Eligible study participants responded to a baseline questionnaire. Three months after the completed intervention, the study participants were sent a follow-up questionnaire (Fig. 1).

### Study-specific questionnaire

The validated study-specific questionnaire consisted of 175 questions including items concerning demographic details, aspects of wellbeing, body image, childbirth, intestinal-, and urinary tract health, sexual function, sexual abuse, and lymphedema. Some questions served to rank the patient’s most distressing symptoms. A self-reported evaluation was included in the follow-up questionnaire. Detailed information on the questionnaire can be found in a previous paper [29].

### Nurse-led interventions

The nurse-led clinic, established in 2011 at Sahlgrenska University Hospital, was based on the national strategy proposals financed by the Regional Cancer Centre in Western Sweden. Currently, the team consists of three clinical oncology nurses, specialized in understanding and addressing pelvic cancer survivorship late effects, and one gynecologic oncologist. The overall goal with the interventions is to provide treatment and support for radiotherapy-induced late effects and chronic states in order to improve quality of life.

The follow-up consisted of visits and/or phone calls or digital meetings focusing on communication to support both physical and psychosocial health challenges. The duration of contact varied from 3 months to several years due to the individualized care and the severity of symptoms that could require varying content and extended interventions. In some cases, a partner was involved. Referrals were sent to experts if necessary. Intestinal health interventions followed an algorithm based on research by our and other research groups [18, 30], further developed and adopted by the Swedish national pelvic cancer healthcare rehabilitation program [31].

Sexual health interventions consisted of both psychoeducational efforts and efforts to provide a broader perspective of sexuality and sexual experiences. Using the PLISSIT model [32], information was provided about common radiotherapy-induced vaginal changes and menopausal symptoms, the consequences of the lack of estrogen that may affect the patient, and infertility. Counseling concerning vaginal dilator therapy, topical estrogen, guidance, and suggestions regarding lack of desire and barriers to intimacy and partner communication were also given.

### Data processing

Data from the questionnaires were coded and transferred to EpiDataSoftware V.3.1 (EpiData Association) and then exported to Microsoft Excel. The first analyses focused on whether there was a change of at least one step along the scale of the baseline questionnaire compared to the follow-up questionnaire, e.g., from “a little” to “moderate” or from “moderate” to “a lot.” Any changes in a positive direction were recognized as clinically meaningful improvement. A sequence of univariate analyses was conducted to explore associations between improvement in intestinal health and improvement in satisfaction with overall sexual health, quality of life, depressed, or anxious mood. To ensure that no misrepresentation regarding respondents versus non-respondents was present, a random sample of 20 non-respondents was selected for a dropout analysis (Fig. 1). According to medical records, six people had died, two could not be reached, and two declined to attend. The remaining ten non-respondents were contacted through first a letter. For ethical reasons, they were requested to send a text message if they declined participation. The participating respondents were contacted by phone and asked to answer eight questions to assess the level of physical and psychosocial health problems at the time of the 3-month follow-up.

### Statistical analysis

Frequency and percentages were used for descriptive statistics. The Wilcoxon signed-rank test was used to assess changes in the variables for sexual health and wellbeing aspects included in the analysis at follow-up compared to baseline. Changes were trichotomized into three categories: increase, no change, and decrease (see Table 2). Subsequently, to assess the associations between change in overall satisfaction with sexual life and wellbeing aspects on the one hand, and changes in bowel symptom intensities at follow-up compared to baseline on the other hand, changes in both classes in the categories “improvement” and “no improvement” were dichotomized. A log-binomial regression model was performed to assess the association between each pair of dichotomized changes in overall sexual health or the wellbeing aspects and dichotomized changes in bowel symptom intensities. Likelihood ratio test *p* values were calculated to assess the level of statistical significance for the estimated effect measures, i.e., relative risks. The level of significance (*p* value) was set to 0.05. All calculations were performed in R version 4.0.0.

## Ethical considerations

The study was approved by the Regional Ethical Committee (EPN) in Gothenburg (D 686–10). Informed consent was obtained from all participants included in the study.

## Results

### Characteristics

A total of 260 female pelvic cancer survivors who had completed both the baseline questionnaire and the 3-month follow-up questionnaire, pre-and post-intervention between 2011 and 2017, were eligible for inclusion in the analysis. Baseline characteristics of the participants are shown in Table 1. The most common diagnosis was endometrial cancer (32.7%), followed by rectal- (25.8%), cervical- (20.8%), anal- (16.9%), and vulvar cancer (2.3%). The majority of the study participants had undergone surgery as a part of their cancer treatment (73.1%). The mean age was 62.5 years, 71.5% of the women were married or living with a partner.

**Table 1:** Demographics and clinical characteristics of study participants

Characteristics	Total study group of female pelvic cancer survivors, *N* = 260
**Cancer type**	*N (%)*
Endometrial cancer	85 (32.7)
Cervical cancer	54 (20.8)
Vulvar cancer	6 (2.3)
Rectal cancer	67 (25.8)
Anal cancer	44 (16.9)
Other	4 (1.5)
**Age in years**	*Mean* ± *SD* 62.5 ± 11.7
**Age, grouped**	*N (%)*
–29	1 (0.4)
30–39	12 (4.6)
40–49	17 (6.5)
50–59	70 (26.9)
60–69	85 (32.7)
70–79	60 (23.1)
80–	13 (5.0)
**Years since radiotherapy**	*Mean* ± *SD* 2.9 ± 4.2
**Years since radiotherapy, grouped**	*N (%)*
0.5	16 (6.2)
1	90 (34.6)
2	39 (15.0)
3	68 (26.2)
> 4	46 (17.6)
**Cancer treatment**	*N (%)*
External radiotherapy with and without brachytherapy	70 (26.9)
Surgery and external radiotherapy with and without brachytherapy	190 (73.1)
**Marital status**	*N (%)*
Married or living with a partner	186 (71.5)
Widow	22 (8.5)
Has a partner but lives alone	13 (5.0)
Single	39 (15.0)
**Education level**	*N* = *259*
Elementary school	55 (21.2)
Secondary school	111 (42.7)
College/university	93 (35.8)
**Employment status**	*N* = *259*
Student	2 (0.8)
Unemployed job-seeker	5 (1.9)
Employed	96 (36.9)
Housewife	1 (0.4)
On sick leave	20 (7.7)
Disability pension	17 (6.5)
Retired	118 (45.4)
**Resident**	
In the countryside	52 (20.0)
Small or medium-sized city	123 (47.3)
In a big city	85 (32.7)
**Smoker**	*N* = *235*
No	204 (86.8)
Yes	31 (13.2)

Number (*n*) and proportion (%) of women is presented. *N* delivering data is presented in case of missing data. *SD*, standard deviation

### Sexual health and wellbeing aspects

We studied changes in levels of satisfaction with overall sexuality and sexual life at baseline vs. 3-month follow-up. As shown in Table 2, a higher proportion of women reported increased rather than decreased satisfaction with overall sexual health and sexual life (26.0% vs. 15.3%, *p* = 0.035). Reduced superficial genital pain (25.8% vs. 13.1%, *p* ≤ 0.025) and reduced deep genital pain during vaginal sex (23.1% vs. 8.0%, *p* ≤ 0.001) were reported post-intervention. More women reported a decrease than an increase in their ability to have an orgasm (19.4% vs. 14.8%, *p* = 0.041), while the majority (65.7%) reported no change in this sexual function following the nurse-led interventions.

**Table 2:** Analysis of changes in aspects of sexual health and wellbeing among female pelvic cancer survivors who completed both the baseline and the 3-month follow-up questionnaire post-intervention

Aspects assessed	*N* (%)	*p* value
**Satisfaction with overall sexual life—baseline versus follow-up**	*N* = *215*	**0.035**
No change	126 (58.6)	
Increased satisfaction with overall sexuality and sexual life	56 (26.0)	
Decreased satisfaction with overall sexuality and sexual life	33 (15.3)	
**Sexual thoughts—baseline versus follow-up**	*N* = *217*	0.097
No change	127 (58.5)	
Increase in frequency of sexual thoughts	32 (14.7)	
Decrease in frequency of sexual thoughts	58 (26.7)	
**Level of sexual thoughts and fantasies—baseline versus follow-up**	*N* = *213*	0.766
No change	138 (64.8)	
Increase in frequency of sexual fantasy	36 (16.9)	
Decrease in frequency of sexual fantasy	39 (18.3)	
**Arousal in sexual situations—baseline versus follow-up**	*N* = *214*	0.264
No change	127 (59.3)	
Increase in frequency of arousal when in sexual situations	39 (18.2)	
Decrease in frequency of arousal when in sexual situations	48 (22.4)	
**Lubrication in sexual situations—baseline versus follow-up**	*N* = *214*	0.147
No change	133 (62.1)	
Increase in frequency of lubrication when in sexual situations	35 (16.3)	
Decrease in frequency of lubrication when in sexual situations	46 (21.5)	
**Noticed genital swelling during sexual arousal—baseline versus follow-up**	*N* = *206*	0.723
No change	149 (72.3)	
Increase in frequency of genital swelling when in sexual situations	27 (13.1)	
Decrease in frequency of genital swelling when in sexual situations	30 (14.6)	
**Orgasm frequency—baseline versus follow-up**	*N* = *217*	0.081
No change	121 (55.8)	
Increase in frequency of orgasm	39 (18.0)	
Decrease in frequency of orgasm	57 (26.3)	
**Ability to have an orgasm during sexual stimulation or vaginal sex—baseline versus follow-up**	*N* = *216*	**0.041**
No change	142 (65.7)	
Increase in the ability to have an orgasm	32 (14.8)	
Decrease in the ability to have an orgasm	42 (19.4)	
**Noticed vaginal shortness during vaginal sex—baseline versus follow-up**	*N* = *220*	0.559
No change	147 (66.8)	
Increased vaginal length when in vaginal sex situations	26 (11.8)	
Decreased vaginal length when in vaginal sex situations	47 (21.4)	
**Noticed vaginal inelasticity during vaginal sex—baseline versus follow-up**	*N* = *213*	0.769
No change	144 (67.6)	
Increased vaginal elasticity when in vaginal sex situations	28 (13.1)	
Decreased vaginal elasticity when in vaginal sex situations	41 (19.2)	
**Numbness in labia or inside of thighs—baseline versus follow-up**	*N* = *186*	0.338
No change	124 (66.7)	
Decrease in numbness in labia/inside of thighs	32 (17.2)	
Increase in numbness in labia/inside of thighs	30 (16.1)	
**Superficial genital pain during vaginal sex—baseline versus follow-up**	*N* = *213*	**0.025**
No change	130 (61.0)	
Decreased level of superficial genital pain during vaginal sex	55 (25.8)	
Increased level of superficial genital pain during vaginal sex	28 (13.1)	
**Deep genital pain during vaginal sex—baseline versus follow-up**	*N* = *212*	** < 0.001**
No change	146 (68.9)	
Decreased level of deep genital pain during vaginal sex	49 (23.1)	
Increased level of deep genital pain during vaginal sex	17 (8.0)	
**Frequency of vaginal sex—baseline versus follow up**	*N* = *206*	0.476
No change	133 (64.6)	
Increase in frequency of vaginal sex	36 (17.5)	
Decrease in frequency of vaginal sex	37 (18.0)	
**Level of quality of life—baseline versus follow-up**	*N* = *246*	
No change	86 (35.0)	** < 0.001**
Increase in level of quality of life	105 (42.7)	
Decrease in level of quality of life	55 (22.4)	
**How often feeling depressed—baseline versus follow-up**	*N* = *246*	
No change	71 (28.9)	**0.003**
Decrease in how often feeling depressed	106 (43.1)	
Increase in how often feeling depressed	69 (28.0)	
**How often feeling worried or anxious—baseline versus follow-up**	*N* = *246*	
No change	73 (29.7)	** < 0.001**
Decrease in how often feeling worried or anxious	113 (45.9)	
Increase in how often feeling worried or anxious	60 (24.4)	

Number (*n*) and proportion (%) of women is presented. *p* values in bold font indicate statistical significance (≤ 0.05). A change of at least one step along the scale in the baseline questionnaire, compared to the follow-up questionnaire was recognized as a clinically meaningful improvement. *N* delivering data is presented in case of missing data

In the analysis of wellbeing aspects, almost twice as many women reported an increased rather than a decreased level of quality of life (42.7% vs. 22.4%, *p* ≤ 0.001) post-intervention (Table 2). A statistically significantly higher proportion of the women reported reduced depressed (43.1% vs. 28.0%, *p* = 0.003) or anxious mood (45.9% vs. 24.4%, *p* ≤ 0.001).

### Improvement in intestinal health

According to the univariable analysis (Table 3), statistically significant associations were found between reduced urgency to defecate and improved satisfaction with overall sexual health (RR 3.12, CI 1.27–7.68, *p* = 0.004) and between reduced urgency to defecate with fecal leakage and reduced anxiety (RR 1.56, CI 1.04–2.33, *p* = 0.021). Although not statistically significant, the following factors seemed slightly associated with improved quality of life: reduced frequency of defecation, defecation urgency, defecation urgency with fecal leakage, leakage volume, and leakage without forewarning. As shown in Table 4, severe intestinal symptoms reduced following the interventions, for example, high frequency (5 times per day or more often) of defecation (6.4% vs. 4.1%), high frequency (at least every day) of loose stool (17.4% vs. 11.3%), unable to hold gas (at least every day) (18.4% vs. 11.3%), urgency (at least every day) to defecation (21.1% vs. 13.4%), unable to hold stool < 1 min (18.6% vs. 11.4%), and defecation urgency with fecal leakage (at least once per week, 3 times per week, at least every day) (summed up, 16.6% vs. 8%).

**Table 3:** Improved intestinal health aspects as explanatory variables for improved level of satisfaction with overall sexual life and wellbeing aspects

	Satisfaction with overall sexuality and sexual health			Quality of life			How often feeling depressed			How often feeling worried or anxious		
	*N* (%)	RR (95% CI**)**	*p* value	*N* (%)	RR (95% CI)	*p* value	*N* (%)	RR (95% CI)	*p* value	*N* (%)	RR (95% CI)	*p* value
Frequency of defecation	No improvement 4/22 (18.2)	Ref. 1.0	0.500	No improvement 6/14 (42.9)	Ref. 1.0	**0.035**	No Improvement 9/15 (60)	Ref. 1.0	0.874	No improvement 10/23 (43.5)	Ref. 1.0	0.611
	Improvement 10/39 (25.6)	1.41 (0.50–3.97)		Improvement 22/29 (75.9)	1.77 (0.93–3.35)		Improvement 19/33 (57.6)	1 (0.60–1.66)		Improvement 22/44 (50)	1.55 (0.66–2.00)	
Frequency of loose stool	No improvement 6/36 (16.7)	Ref. 1.0	0.418	No improvement 16/27 (59.3)	Ref. 1.0	0.962	No improvement 15/29 (51.7)	Ref. 1.0	0.255	No improvement 20/41 (48.8)	Ref. 1.0	0.693
	Improvement 17/73 (23.3)	1.4 (0.60–3.24)		Improvement 27/46 (58.7)	1 (0.71–1.42)		Improvement 38/59 (64.4)	1.25 (0.84–1.86)		Improvement 36/80 (45)	1 (0.67–1.49)	
Unable to hold gas when needed	No improvement 5/22 (22.7)	Ref. 1.0	0.812	No improvement 16/20 (80)	Ref. 1.0	0.431	No improvement 11/19 (57.9)	Ref. 1.0	0.434	No improvement 14/27 (51.9)	Ref. 1.0	0.716
	Improvement 6/30 (20)	1 (0.35–2.86)		Improvement 16/23 (69.6)	1 (0.71–1.42)		Improvement 18/26 (69.2)	1.2 (0.75–1.90)		Improvement 17/36 (47.2)	1 (0.61–1.65)	
Urgency to defecate	No improvement 5/46 (10.9)	Ref. 1.0	**0.004**	No improvement 24/37 (64.9)	Ref. 1.0	0.744	No improvement 17/34 (53.1)	Ref. 1.0	0.191	No improvement 23/51 (45.1)	Ref. 1.0	0.899
	Improvement 20/59 (33.9)	**3.12 (1.27–7.68)**		Improvement 26/38 (68.4)	1.05 (0.77–1.45)		Improvement 37/55 (67.3)	1.27 (0.87–1.84)		Improvement 31/67 (46.3)	1.03 (0.69–1.53)	
For how long able to hold stool	No improvement 22/102 (21.6)	Ref. 1.0	0.369	No improvement 49/74 (66.2)	Ref. 1.0	0.675	No improvement 49/76 (64.5)	Ref. 1.0	0.373	No improvement 55/113 (48.7)	Ref. 1.0	0.285
	Improvement 18/65 (27.7)	1.28 (0.75–2.20)		Improvement 30/48 (62.5)	1 (0.76–2.31)		Improvement 33/58 (56.9)	1 (0.76–1.32)		Improvement 31/76 (40.8)	1 (0.72–1.39)	
Urgency to defecate with fecal leakage	No improvement 11/53 (20.8)	Ref. 1.0	0.393	No improvement 18/31 (58.1)	Ref. 1.0	0.117	No improvement 18/34 (52.9)	Ref. 1.0	0.064	No improvement 20/57 (35.1)	Ref. 1.0	**0.021**
	Improvement 21/77 (27.3)	1.31 (0.69–2.49)		Improvement 46/62 (74.2)	1.28 (0.92–1.78)		Improvement 48/67 (71.6)	1.35 (0.95–1.1.92)		Improvement 47/86 (54.7)	**1.56 (1.04–2.33)**	
Leakage volume	No improvement 13/59 (22)	Ref. 1.0	0.658	No improvement 20/34 (58.8)	Ref. 1.0	0.082	No improvement 27/42 (64.3)	Ref. 1.0	0.758	No improvement 27/63 (42.9)	Ref. 1.0	0.533
	Improvement 18/71 (25.4)	1.15 (0.62–2.15)		Improvement 42/55 (76.4)	1.3 (0.95–1.78)		Improvement 39/58 (67.2)	1.05 (0.78–1.40)		Improvement 36/79 (48.1)	1.12 (0.78–1.62)	
Leakage without forewarning		N/A*		No improvement 1/2 (50)	Ref. 1.0	0.352	No improvement 3/6 (50)	Ref. 1.0	0.455	No improvement 3/8 (37.5)	Ref. 1.0	0.683
				Improvement 21/26 (80.8)	1.62 (0.40–6.54)		Improvement 16/24 (66.7)	1.33 (0.57–3.12)		Improvement 15/33 (45.5)	1.21 (0.46–3.20)	

Number (*N*) and proportion (%) of women is presented. Relative risk (RR), reference category (Ref.), confidence interval (CI). *p* values in bold font indicate statistical significance (≤ 0.05). *Not applicable (N/A). At least one step of change in a positive direction from baseline to follow-up, in each aspect assessed, was considered an improvement

**Table 4:** Frequencies and proportions of self-reported aspects of sexual health, wellbeing, and intestinal symptoms pre- and post-intervention

	Categories	Pre-intervention *N* (%)	Post-intervention *N* (%)
**Sexual health**			
		*N* = *247*	*N* = *224*
Feeling of sexual attractiveness	Not at all	153 (61.9)	120 (53.6)
	A little	51 (20.6)	49 (21.9)
	Moderate	35 (14.2)	47 (21.0)
	A lot	8 (3.2)	8 (3.6)
		*N* = *246*	*N* = *221*
Sexual thoughts, frequency	Never	84 (34.1)	95 (43.0)
	A few times	116 (47.2)	90 (40.7)
	Every month	23 (9.3)	4 (1.8)
	Every week	20 (8.1)	24 (10.9)
	Every day	3 (1.2)	8 (3.6)
		*N* = *223*	*N* = *203*
If thoughts of sex persist, how satisfied	Not at all	76 (34.1)	57 (28.1)
	A little	47 (21.1)	42 (20.7)
	Moderate	53 (23.8)	51 (25.1)
	A lot	47 (21.1)	53 (20.4)
		*N* = *242*	*N* = *218*
Level of sexual thoughts and fantasies	No level at all	78 (32.2)	78 (35.8)
	Low level	119 (49.2)	97 (44.5)
	Moderate level	41 (16.9)	37 (17.0)
	High level	4 (1.7)	6 (2.8)
		*N* = *241*	*N* = *219*
Arousal in sexual situations	Not relevant	116 (48.1)	111 (50.7)
	Never	17 (7.1)	15 (6.8)
	Less than one of five times	23 (9.5)	18 (8.2)
	Less than half of the times	11 (4.6)	8 (3.7)
	About half of the times	16 (6.6)	16 (7.3)
	More than half of the times	18 (7.5)	13 (5.9)
	Every time	40 (16.6)	38 (17.4)
		*N* = *241*	*N* = *220*
Lubrication in sexual situations	Not relevant	118 (49.0)	114 (51.8)
	Never	24 (10)	20 (9.1)
	Less than one of five times	17 (7.1)	17 (7.7)
	Less than half of the times	9 (3.7)	8 (3.6)
	About half of the times	19 (7.9)	14 (6.4)
	More than half of the times	15 (6.2)	15 (6.8)
	Every time	39 (16.2)	32 (14.5)
		*N* = *242*	*N* = *219*
Lubrication sufficient for vaginal sex	Not relevant	127 (52.5)	120 (54.8)
	Never sufficient	34 (14.0)	32 (14.6)
	Frequently reduced/insufficient	31 (12.8)	18 (8.2)
	Sometimes reduced/insufficient	31 (12.8)	33 (15.1)
	Always sufficient	19 (7.9)	16 (7.3)
		*N* = *235*	*N* = *215*
Noticed genital swelling during sexual arousal	Not relevant	141 (60.0)	129 (60.0)
	Less than one of five times	23 (9.8)	18 (8.4)
	Less than half of the times	12 (5.1)	8 (3.7)
	About half of the times	14 (6.0)	18 (8.4)
	More than half of the times	17 (7.2)	20 (9.3)
	Every time	28 (11.9)	22 (10.2)
		*N* = *246*	*N* = *219*
Orgasm frequency	Not relevant	106 (43.1)	107 (48.9)
	Never	37 (15.0)	28 (12.8)
	A few times	43 (17.5)	28 (12.8)
	1–2 times per month	24 (9.8)	16 (7.3)
	3–4 times per month	18 (7.3)	24 (11.0)
	1–2 times per week	16 (6.5)	13 (5.9)
	< 2 times per week	2 (0.8)	3 (1.9)
		*N* = *245*	*N* = *219*
How easy orgasm during sexual stimulation or vaginal sex	Not relevant	118 (48.2)	113 (51.6)
	Very easy	15 (6.1)	7 (3.2)
	Easy	54 (22)	53 (24.2)
	Difficult	42 (17.1)	35 (16.0)
	Very difficult	16 (6.5)	11 (5.0)
		*N* = *246*	*N* = *216*
Noticed vaginal shortness during vaginal sex	Not relevant	134 (54.5)	112 (51.9)
	Not at all	39 (15.9)	32 (14.8)
	A little	20 (8.1)	28 (13.0)
	Moderate	16 (6.5)	13 (6.0)
	A lot	37 (15.0)	31 (14.4)
		*N* = *244*	*N* = *217*
Noticed vaginal inelasticity during vaginal sex	Not relevant	135 (55.3)	115 (53.0)
	Not at all	34 (13.9)	27 (12.4)
	A little	29 (11.9)	30 (13.8)
	Moderate	14 (5.7)	14 (6.5)
	A lot	32 (13.1)	31 (14.3)
		*N* = *240*	*N* = *196*
Level of distress if vaginal shortness or inelasticity persists	Not relevant	136 (56.7)	135 (68.9)
	Not at all	5 (2.1)	18 (9.2)
	A little	9 (3.8)	19 (9.7)
	Moderate	29 (12.1)	6 (3.1)
	A lot	61 (25.4)	18 (9.2)
		*N* = *242*	*N* = *196*
Numbness in labia or inside of thighs	Never	158 (68.1)	135 (68.9)
	Seldom	14 (6.0)	18 (9.2)
	Sometimes	25 (10.8)	19 (9.7)
	Often	18 (7.8)	6 (3.1)
	Always	17 (7.3)	18 (9.2)
		*N* = *244*	*N* = *217*
Superficial genital pain during vaginal sex	Not relevant	144 (59.0)	131 (60.4)
	Not at all	31 (12.7)	29 (13.4)
	A little	24 (9.8)	30 (13.8)
	Moderate	22 (9.0)	12 (5.5)
	A lot	23 (9.4)	15 (6.9)
		*N* = *243*	*N* = *217*
Deep genital pain during vaginal sex	Not relevant	146 (60.1)	142 (65.4)
	Not at all	48 (19.8)	44 (20.3)
	A little	17 (7.0)	15 (6.9)
	Moderate	13 (5.3)	8 (3.7)
	A lot	19 (7.8)	8 (3.7)
		*N* = *241*	*N* = *210*
Frequency of vaginal sex	Never	159 (66.0)	136 (64.8)
	A few times	31 (12.9)	19 (9.0)
	1–2 times per month	21 (8.7)	24 (11.4)
	3–4 times per month	17 (7.1)	18 (8.6)
	1–2 times per week	11 (4.6)	8 (3.8)
	< 2 times per week	2 (0.8)	5 (2.4)
		*N* = *243*	*N* = *221*
Overall satisfaction with sexual life	Not relevant	93 (38.3)	84 (38.0)
	Not at all	80 (32.9)	53 (24.0)
	A little	24 (9.9)	24 (10.9)
	Moderate	29 (11.9)	36 (16.3)
	A lot	17 (7.0)	24 (10.9)
**Wellbeing**			
		*N* = *258*	*N* = *247*
Level of QoL^a^	No QoL at all or very low	22 (8.5)	9 (3.6)
	Moderate QoL	175 (67.3)	159 (64.4)
	Very high	61 (23.5)	79 (32)
		*N* = *258*	*N* = *248*
How often feeling depressed	Always	27 (8.5)	22 (8.9)
	Sometimes	143 (55.4)	119 (48.0)
	Never	88 (34.1)	107 (43.1)
		*N* = *258*	*N* = *248*
How often feeling worried or anxious	Always	34 (13.2)	26 (10.5)
	Sometimes	138 (53.5)	113 (45.6)
	Never	86 (33.3)	109 (44.0)
**Intestinal function**			
		*N* = *250*	*N* = *241*
Frequency of defecation	Every other day	26 (10.4)	21 (8.7)
	Less than every other day	14 (5.6)	18 (7.7)
	Once per day	31 (12.4)	35 (14.5)
	1–2 times per day	49 (19.6)	68 (28.2)
	2–3 times per day	58 (23.2)	53 (22.0)
	3–4 times per day	56 (22.4)	36 (14.9)
	5 times per day or more often	16 (6.4)	10 (4.1)
		*N* = *253*	*N* = *248*
Frequency of loose stool	No	34 (13.4)	62 (25.0)
	Occasionally	60 (23.7)	76 (30.6)
	At least once per month	29 (11.5)	23 (9.3)
	At least once per week	47 (18.6)	38 (15.3)
	At least 3 times per week	39 (15.4)	21 (8.5)
	At least every day	44 (17.4)	28 (11.3)
		*N* = *248*	*N* = *234*
Flatulence	No self-perception of odor	99 (39.9)	112 (47.9)
	Self-perception of odor	149 (57.3)	122 (52.1)
		*N* = *245*	*N* = *231*
Unable to hold gas when needed	No	53 (21.6)	66 (28.6)
	Occasionally	72 (29.4)	89 (38.5)
	At least once per month	23 (9.4)	6 (2.6)
	At least once per week	27 (11.0)	24 (10.4)
	At least 3 times per week	25 (10.2)	20 (8.7)
	At least every day	45 (18.4)	26 (11.3)
		*N* = *245*	*N* = *238*
Urgency to defecation	No	42 (17.1)	52 (21.8)
	Occasionally	49 (19.9)	85 (35.7)
	At least once per month	27 (11.0)	14 (5.9)
	At least once per week	40 (16.3)	30 (12.6)
	At least 3 times per week	35 (14.2)	25 (10.5)
	At least every day	52 (21.1)	32 (13.4)
		*N* = *253*	*N* = *246*
For how long able to hold stool	Not relevant, having a stoma	38 (15)	42 (17.1)
	< 1 min	47 (18.6)	28 (11.4)
	1–5 min	94 (37.2)	82 (33.3)
	5–10 min	38 (15.0)	42 (17.1)
	10–30 min	21 (8.3)	33 (13.4)
	> 30 min	15 (5.9)	19 (7.7)
		*N* = *247*	*N* = *238*
Defecation urgency with fecal leakage	No	93 (37.7)	135 (56.7)
	Occasionally	95 (38.5)	70 (29.4)
	At least once per month	18 (7.3)	14 (5.9)
	At least once per week	24 (9.7)	10 (4.2)
	At least 3 times per week	12 (4.9)	4 (1.7)
	At least every day	5 (2.0)	5 (2.1)
		*N* = *244*	*N* = *239*
Leakage volume	Not relevant	91 (37.3)	138 (57.7)
	Soiling	76 (31.1)	50 (20.9)
	A small volume	53 (21.7)	42 (17.6)
	A large volume	21 (8.6)	8 (3.3)
	The complete volume	3 (1.2)	1 (0.4)
		*N* = *246*	*N* = *237*
Leakage without forewarning	No	145 (58.9)	171 (72.2)
	Occasionally	75 (30.5)	51 (21.5)
	At least once per month	12 (4.9)	5 (2.1)
	At least once per week	8 (3.3)	3 (1.3)
	At least 3 times per week	3 (1.2)	2 (0.8)
	At least every day	3 (1.2)	5 (2.1)
		*N* = *248*	*N* = *239*
Leakage of the total volume without forewarning	No	205 (82.7)	222 (92.9)
	Occasionally	34 (13.7)	11 (4.6)
	At least once per month	4 (1.6)	4 (1.7)
	At least once per week	3 (1.2)	2 (0.8)
	At least 3 times per week		
	At least every day	2 (0.8)	
		*N* = *250*	*N* = *241*
Feeling embarrassed due to fecal leakage	Not relevant	76 (30.4)	126 (52.3)
	No	92 (36.8)	75 (31.1)
	A little	47 (18.8)	21 (8.7)
	Moderate	16 (6.4)	14 (5.8)
	A lot	19 (7.6)	5 (2.1)
		*N* = *248*	*N* = *243*
Fecal leakage hinders sexual life	Not relevant	153 (61.7)	183 (75.3)
	No	75 (30.2)	52 (21.4)
	Yes	20 (8.1)	8 (3.3)
		*N* = *248*	*N* = *245*
Fecal leakage affects quality of life	Not relevant	88 (35.5)	114 (46.5)
	Not at all	37 (14.9)	40 (16.3)
	A little	59 (23.8)	51 (20.8)
	Moderate	34 (13.7)	20 (8.2)
	A lot	30 (12.1)	20 (8.2)

*N*, numbers; *QoL*, quality of life. ^a^Patient-reported answers with the range 1–7 and classified as follows: 1–2 No QoL at all or very low, 3–5 “Moderate,” and 6–7 “High.” ^b^Patient-reported answers with the range 1–7 classified as follows: 1–2 “Never,” 3–5 “Sometimes,” and 6–7 “Always.” *N* delivering data are presented in case of missing data

### Proportions of symptoms pre- and post-intervention

Although no statistically significant comparisons were made when analyzing proportions of answers pre- and post-interventions, we found reports of slightly decreased symptoms of genital pain, depression, anxiety, and almost all intestinal health variables post-intervention (Table 4). Women with a high degree of distress from persistent vaginal shortness and inelasticity were lower post-intervention.

### Evaluation of interventions

**Fig. 2:**
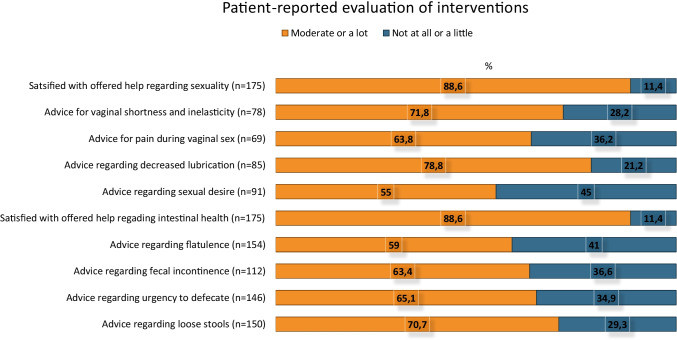
Number (*n*) and proportions (%) of patient-reported evaluation, 3 months post-interventions

The majority of the respondents (88.6%) reported that they were moderately to very satisfied with help offered regarding sexual health issues and intestinal symptoms (Fig. 2). Advice received for late effects had been moderately to very beneficial to the majority of women.

## Discussion

The findings from this study suggest that sexual health and wellbeing can improve in women treated with pelvic radiotherapy through interventions provided in a nurse-led clinic focusing on a wide range of physical treatment-induced late effects. Specifically, quality of life, satisfaction with overall sexual health, and decrease in pain during vaginal sex were statistically significantly improved. Regarding intestinal health, reduced episodes of defecation were statistically significantly associated with improved satisfaction with overall sexual health. Moreover, we found reduced urgency to defecate associated with reduced degree of anxiety. Regarding sexual function, improvement was not found in all aspects. For the majority of the women, vaginal shortness and inelasticity did not improve, nor did lubrication, genital swelling, or the ability to achieve orgasm.

To the best of our knowledge, no other studies have taken a similar approach that is studying sexual health in women with a wide range of problems, providing multidimensional individualized interventions directed at women with a history of pelvic radiotherapy, and mainly focusing on physical treatment-induced late effects but with a psychosocial approach. In a recent study among elderly individuals, an increase was observed in the number of physical illnesses, which associated with a decrease in satisfaction with sexual life [33], a finding similar to the results in the current study. In previous research, gynecological cancer survivors with fecal incontinence reported low quality of life [10]. A recent study reported that fecal incontinence entails a constant uncertainty regarding leakage and dealing with shame and degradation in social situations [23]. We found that improvements in intestinal symptoms could increase women’s quality of life and sexual wellbeing, and lead to lower levels of anxiety. Thus, it seems important to prioritize individuals’ physical illness in parallel with the initiation of sensitive and individualized discussions both early and later in the rehabilitation process, since questions about sexuality may arise when other troublesome symptoms have been successfully treated. While depression and anxiety are previously shown to be more prevalent among women with a history of sexual abuse [7, 34], this also needs to be considered in counseling.

A high prevalence of genital pain and vaginal shortness among women treated with pelvic radiotherapy has been previously reported [7, 8, 16]. Since interventions provided in the current study could explain the decrease in genital pain, we suggest that available guidelines [14, 35], including local estrogen, lubricants, discussing how to carry out vaginal dilator therapy, and alternative ways to practice sex, could be more frequently used in clinical practice. However, it is known that genital pain may include underlying causes, such as decreased sexual desire and experiencing pain instead of pleasure. One study reported that some (young) women prioritize their partner’s enjoyment before their own, despite pain [36], which may also be relevant for cancer survivors and needs to be taken into account in the support of female pelvic cancer survivors as well. Furthermore, in the light of the previously reported relationship between genital pain and experience of sexual abuse [7, 34], this also needs to be discussed in counseling, however not further explored in the current paper. As previously mentioned, time spent educating, discussing, and supporting are vital parts of counseling given in addition to medication or devices [24, 25, 28]. When it comes to vaginal changes, the irreversible effect of radiotherapy on vaginal epithelial tissue [8, 37], nerves, and vessels [38, 39] probably explains the lack of improvement in vaginal changes and sexual function in our study. These late effects could be addressed, not only by vaginal dilator therapy and individual counseling, but also by preventative planning of organ-sparing radiotherapy.

Previous research reports that patients want healthcare professionals to raise the topic of sexuality [40]. Although the balance between providing too much or too little information before cancer treatment is challenging, early information could improve women’s preparedness for possible sexual side effects. In a previous study, low preparedness for sexual side effects post-surgery in prostate cancer survivors was reported to negatively influence the bother of sexual side effects and negatively affect self-esteem [41], which may also apply to female pelvic cancer survivors. In our opinion and as previously suggested, oncology nurses are well-placed to implement and develop cancer prehabilitation interventions [42] and could offer a solution to the widespread issue of late effects following pelvic radiotherapy.

### Strengths and limitations

Strengths of this study include the large population-based cohort, the longitudinal design, the novelty of the comprehensive nurse-led interventions targeting female pelvic cancer survivors, and the use of a validated pelvic cancer-specific questionnaire. However, the questionnaire was validated for female pelvic survivors but not for survivors invited to undergo interventions. Hence, one could argue that the reliability may be limited since the participants were not anonymous, which could bias the responses. The fact that the women were recruited from only the western region in Sweden might induce generalization problems. However, this region represents 1/5 of the Swedish population. Nevertheless, we do not know if the results in this study are applicable to other populations. We consider data prior to pelvic radiation would have facilitated comparisons of health aspects pre- and post-cancer treatment. The diverse treatment schedules due to the various cancer diagnoses and differences in time since completed cancer treatment may be considered limitations. Furthermore, women referred to the clinic might report more severe late effects compared to the inventoried patients, possibly diluting the effects in our analysis. According to the dropout analysis, no differences in the magnitude of symptoms between the respondents and the non-respondents were recognized.

Except for the biometric data, all data used in the current study are “recalled”; thus, recall bias could be present. As with any single-arm prospective study, our results may be influenced by selection bias, more specifically that women with a high magnitude of radiotherapy-induced late effects may be more likely to take part in the interventions. In future studies, evaluating the impact of baseline patient characteristics, including age, previous sexual abuse, and side effects beyond sexual- and intestinal dysfunctions, will be of interest.

### Conclusions

We conclude that poor quality of life and low levels of satisfaction with sexual health among female pelvic cancer survivors can be substantially improved through individualized interventions provided in a nurse-led clinic focusing on physical radiotherapy-induced late effects and diseases. Both prehabilitation and rehabilitation of radiotherapy-induced physical late effects remain a great challenge in cancer care and more intervention studies are needed. In our opinion, oncology nurses with knowledge in medical, psychosocial, and sexual issues are well-placed and well-suited to lead cancer rehabilitation. Our data show the importance of healthcare professionals identifying the needs for support concerning sexual health issues at the start of treatment of pelvic cancer. Although treatment-induced late effects are to some extent inevitable, our data highlight the importance of initiating a careful discussion to establish preparedness for the side effects that may occur. Moreover, there is a need to advance the research in the area of sexual health rehabilitation to optimize treatment strategies to achieve improved sexual health.

## Data Availability

The datasets generated during and analyzed during the current study are available from the corresponding author on reasonable request.
